# Changes in the sodium content of New Zealand packaged breads: 2013 to 2023

**DOI:** 10.1017/jns.2025.10020

**Published:** 2025-07-23

**Authors:** Maria Norburg Tell, Leanne Young, Kathryn Bradbury, Ella Risbrook, Helen Eyles

**Affiliations:** 1 Bra Liv Öxnehaga Primary Health Care Centre, Region Jönköping County, Jönköping and Department of Health, Medicine and Caring Sciences, Linköping University, Linköping, Sweden; 2 Heart Foundation Senior Fellow (HE and KEB), Department of Epidemiology and Biostatistics and The Centre for Translational Health Research, University of Auckland, Auckland, New Zealand; 3 University of Auckland, Auckland, New Zealand

**Keywords:** Bread, New Zealand, Salt, Sodium, WHO, World Health Organization, HSR, Health Star Rating, HF, NZ Heart Foundation, NIP, Nutrition Information Panel, SD, Standard Deviation

## Abstract

We aimed to compare the mean sodium content of New Zealand (NZ) packaged breads in 2013 and 2023 and assess compliance with the NZ Heart Foundation (HF) and World Health Organization (WHO) sodium reduction benchmarks. Sodium data were obtained from a supermarket food composition database. Mean differences between years were assessed using independent samples t-tests and chi-square tests. There was a significant reduction in the sodium content of all bread from 2013 (n=345) to 2023 (n=309) of 46 mg/100g (p<0.001). In 2013, 20% (n=70/345) of breads met the HF benchmarks, and 10% (33/345) met the WHO benchmarks; corresponding values for 2023 were 45% (n=138/309) and 18% (n=57/309) (p<0.001 for both). If continued, the modest reduction in sodium content and increase in the percentage of NZ breads meeting relevant sodium reduction benchmarks could positively affect public health, particularly if extended across the packaged food supply.

## Introduction

Excessive sodium intake is linked to high blood pressure (BP) and non-communicable diseases, including stroke, heart disease, and gastric cancer.^(1,2)^ In 2013, the World Health Organization (WHO) recommended that countries reduce their average population salt intake by 30% toward the upper limit of 5 g per day (2,000 mg sodium)^(3)^ to lower elevated BP by 25% by 2025.^(4)^ Despite NZ’s commitment and high sodium intake (NZ adults consume 3,386 mg per day),^(5)^ no formal sodium reduction programme exists.^(6)^

To support countries in developing a national sodium reduction programme the WHO promotes an evidence-based package of five interventions known as SHAKE (Surveillance, Harness Industry, Adopt Standards for Labelling and Marketing, Knowledge, and Environment).^(7)^ A key component of the SHAKE package is Harnessing Industry to reformulate or reduce sodium in packaged foods. Reformulation is cost-effective,^(8–10)^ equitable, does not require behaviour change,^(11)^ and if done slowly, is well-accepted by consumers.^(12,13)^

While NZ does not currently have a formal salt reduction programme it does have two voluntary reformulation strategies 1) the Health Star Rating (HSR) front-of-pack nutrition label, a scoring system ranging from ½ (least healthy) to five (healthiest) stars^(14)^ which also encourages healthier consumer choice, and 2) the Heart Foundation’s (HF) food industry reformulation programme which sets sodium reduction benchmarks for widely commonly consumed packaged foods^(15)^, lowering them over time according to the food supply’s sodium content.^(16)^

From 2013 to 2019, NZ food products displaying the HSR had a 4% relative decline in sodium.^(17)^ However, the HSR is voluntary and, in 2024, was only present on 33% of eligible supermarket foods,^(18)^ considerably limiting its potential to improve population sodium intake through reformulation or consumer food choice.^(19)^ The HF programme has resulted in a 20 to 25% reduction in the sodium content of top-selling NZ breads (and reformulation of sodium and sugar in other categories),^(20)^ but on its own, it is not an intensive enough intervention to drop population sodium intakes to the WHO target of 2,000 mg/day.^(21)^

In 2021, in the context of limited global action toward population sodium reduction, the WHO created global sodium reduction benchmarks for major packaged food categories^(3)^; supporting the SHAKE package;^(7)^ the benchmarks are stricter than those of the HF, so if implemented, will produce larger population sodium intake reductions. However, there is a lack of published data on how major dietary contributors to population sodium intake are tracking toward either set of targets.

In NZ, bread is the major staple food, with the most recent national intake data (2008/09) showing that bread contributes 11% to the energy intakes of adults; it is also the greatest contributor to dietary sodium (18%).^(22)^ Leavened breads are the most commonly available breads in NZ, with previous research indicating their sodium content has declined e.g. by 14% from 2003 to 2013^(23)^ and 7% from 2007 to 2010.^(24)^ However, recent changes have not been assessed.

## Aims

Our primary aim was to compare the mean sodium content of NZ packaged breads in 2013 and 2023. The secondary aim was to assess the compliance of NZ breads with the NZ HF and WHO sodium reduction benchmarks.

## Methods

Data on the sodium content of NZ packaged breads were taken from the Nutritrack supermarket database (2013 and 2023).^(25)^ Data on the content of lower-sodium salt substitutes in NZ breads were not included because their use in food products and as a replacement for discretionary salt in NZ is anecdotally low. Nutritrack surveys, conducted annually, involve trained personnel collecting data in four leading NZ supermarket chains: New World, Four Square, Woolworths and PAK’nSAVE. Fieldworkers use a customised smartphone app to photograph all products displaying a Nutrition Information Panel (NIP), capturing the product name, brand, nutrition labelling, ingredients and nutrition content. Data from photographs are entered into an online, searchable, web database and categorised into a standardised system.^(26)^

First, breads in Nutritrack with missing or nonsensical sodium data were excluded (Appendix 1). The remaining products were organised into categories defined in the HF and WHO sodium benchmark documents (Table 1). Products not aligning with any category definition were removed. Each categorisation system had different category definitions although there was considerable overlap i.e. the HF system included (1) ‘Leavened bread’ and (2) ‘Flat bread’, based on the presence or absence of yeast, baking soda, or sourdough; the WHO system included (1) ‘Sweet and Raisin bread’ based on presence of fruits or berries and (2) ‘Leavened bread’ and (3) ‘Flat bread’ following the same criteria as for HF; and the Nutritrack system included (1) ‘Fruit bread’ with the same definition as for WHO ’Sweet & raisin bread’, (2) ‘Flatbread’ with the same definition as for WHO and HF, (3) ’Gluten-free flat bread’ (4), ’Gluten-free leavened bread’, (5) ‘Wholemeal bread’, (6) ’Mixed grain bread’, (7) ‘White bread’ and (8) ‘Other bread’ (Table 1).

**Table 1.** Sodium content (mg/100 g) in packaged breads, overall and by bread category, in 2013 and 2023

Bread category		Measures Number of products (n) Mean and median concentration of Sodium in bread (mg/100g)	Year 2013 Sodium content in bread	Year 2023 Sodium content in bread
All bread		n	345	309
		Mean (SD)	440 (126)	394 (128)
		Median (range)	435 (5–842)	383 (2–1070)
HF bread categories	Leavened bread	n	261	196
		Mean (SD)	436 (97)	381 (97)
		Median (range)	432 (5–785)	380 (2–590)
	Flat bread	n	84	113
		Mean (SD)	452 (190)	417 (168)
		Median (range)	441 (6–842)	400 (16–1070)
WHO bread categories	Leavened bread	n	245	186
		Mean (SD)	443 (91)	387 (97)
		Median (range)	438 (5–785)	382 (2–590)
	Flat bread	n	84	112
		Mean (SD)	452 (190)	416 (168)
		Median (range)	441 (6–842)	400 (16–1070)
	Sweet & raisin bread	n	16	11
		Mean (SD)	323 (121)	296 (53)
		Median (range)	350 (11–496)	303 (210–367)
Nutritrack bread categories	Wholemeal bread	n	16	11
		Mean (SD)	414 (35)	406 (53)
		Median (range)	424 (350–450)	380 (365–539)
	Mixed grain bread	n	78	61
		Mean (SD)	430 (47)	374 (48)
		Median (range)	421 (360–582)	378 (200–540)
	White bread	n	35	20
		Mean (SD)	456 (73)	409 (80)
		Median (range)	426 (390–635)	404 (130–546)
	Fruit bread	n	13	10
		Mean (SD)	296 (115)	298 (56)
		Median (range)	325 (11–490)	307 (210–367)
	Flatbread	n	70	83
		Mean (SD)	453 (193)	429 (163)
		Median (range)	441 (6–842)	388 (16–1070)
	Gluten free flatbread	n	1	9
		Mean (SD)	348 (−)	325 (270)
		Median (range)	348 (−)	400 (62–847)
	Gluten free Leavened bread	n	32	20
		Mean (SD)	399 (96)	402 (95)
		Median (range)	385 (242–630)	382 (280–589)
	Other bread	n	100	95
		Mean (SD)	469 (128)	387 (128)
		Median (range)	493 (5–785)	387 (2–645)

Descriptive analyses were undertaken in SPSS 27.0.0, including the mean (standard deviation (SD)), and median (range) sodium content (mg/100g) for all products in 2013 and 2023 and separately for each of the HF, WHO, and Nutritrack categories. Where sufficient data (≥30 products per category for both years) existed, the mean difference in sodium content between years was assessed using independent t-tests for continuous variables (p<0.05). Counts and percentages of products meeting the 2024 HF^(26)^ and 2021 WHO^(3)^ targets current at the time of the research were evaluated separately for each year, and differences were assessed using the chi-square test (p<0.05). No corrections were made for multiple comparisons. A sensitivity analysis was also completed, including only breads available for sale in both years, thus investigating potential reformulation.

## Results

The mean (SD) and median (range) sodium content of packaged breads in 2013 (n=345) and 2023 (n=309) are shown in Table 1. In 2013, the categories with the highest mean sodium content were ‘Flatbread’ (n=84, 452 mg/100g) for both the HF and WHO systems, and ‘Other bread’ (n=100, 469 mg/100g) for the Nutritrack system. In 2023, the categories with the highest mean sodium content were ‘Flatbread’ ((417 mg/100g (n=113) for the HF, 416 mg/100g (n=112) for the WHO and 429 mg/100g (n=83) for Nutritrack). In 2013, the categories with the lowest mean sodium content were ‘Leavened bread’ (436 mg/100g (n=261) for the HF and 443 mg/100g (n=245) for the WHO; and ‘Gluten-free leavened bread’ (399 mg/100g (n=32) for Nutritrack). In 2023, the categories with the lowest mean sodium content were ‘Leavened bread’ (381 mg/100g (n=196) for the HF and 387 mg/100g (n=186) for the WHO; and ‘Mixed grain bread’ at 374 mg/100g (n=61) for Nutritrack).

**Figure 1.**
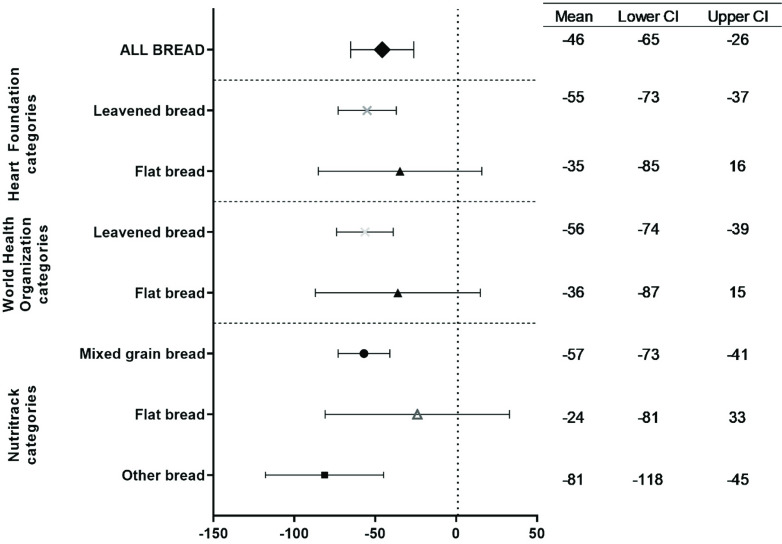
Mean difference (95% CI) in the sodium content of bread by category^1^; between 2013 and 2023. ^1^The following bread categories are not presented as separate categories due to small product numbers (<30 for at least one year) making significance testing inappropriate: Nutritrack categories ‘Wholemeal bread’, ‘White bread’, ‘Fruit bread’, ‘Gluten-free flatbread’ and ‘Gluten-free Leavened bread’ and the WHO category ‘Sweet & raisin bread’. All categories are included in ‘All bread’.

Over the ten years, the overall mean sodium content significantly decreased by 46 mg/100g (p<0.001). Significant reductions over time were also seen in four of the seven categories with sufficient data, i.e. ‘Leavened bread’ (HF and WHO), ‘Mixed grain bread’ and ‘Other bread’, but not ‘Flatbread’ (Fig. 1).

Ninety (identical) breads were available for sale in both 2013 and 2023; the mean (SD) sodium content decreased from 422 mg (SD = 120) in 2013 to 384 mg (SD = 97) in 2023 (p<0.001).

In 2013, 20% (n=70/345) of all breads met the 2024 HF benchmarks, and 10% (n=33/345) met the WHO benchmarks, with significantly more products meeting the benchmarks over time (45% (n=138/309) for the HF and (18% (n=57/309) for the WHO (Appendix 2). Significantly more breads met the benchmarks for three of the seven categories with sufficient data i.e. ‘HF Leavened bread’, ‘WHO Leavened bread’ and ‘Nutritrack mixed grain bread’ (Appendix 2 and Appendix 3).

## Discussion

We found a 10% decrease in the overall mean sodium content of NZ packaged breads from 2013 to 2023, with more products meeting HF and WHO sodium benchmarks over time. The mean sodium content of leavened bread decreased, but there was no significant change in other bread categories.

Our findings align with previous research for earlier timeframes undertaken by Dunford et al. (2007 to 2010)^(24)^ and Monro et al. (for 2003 to 2013),^(23)^ suggesting that efforts to reduce sodium in NZ packaged bread have been ongoing since 2003. Similarly, in the UK, the sodium content of bread and bakery products dropped between 2006 and 2011.^(27)^

However, there is a lack of recent data on bread intake or sales volumes, which is an important limitation hindering an evaluation of the impact of sodium reduction in bread on population sodium consumption. Nonetheless, market data indicate that while the per capita average volume of bread declined from 2018 to 2022, it is now on the rise, highlighting the importance of sodium reduction in bread for lowering population sodium intake in NZ.^(28)^

A key strength of our study was the use of systematically collected data from major NZ supermarkets covering ∼75% of the market.^(29)^ The finding that sodium had reduced in all breads was consistent with the finding that sodium had reduced in breads available for sale in both years, indicating reformulation by food manufacturers had occurred over the 10-year time frame; this is supported by the fact that 62% (56/90) of breads available for sale in both years had decreased in sodium (17% (15/90) increased and 21% (19/90) had not changed).

The observed decline is encouraging; however, the mean decrease of 46 mg per 100g (∼12 mg per slice) is modest in terms of clinical significance and public health impact. An average NZ adult consumes ∼960 kJ of energy from bread or ∼2.5 slices of bread per day.^(30)^ Therefore, the mean decrease in sodium from bread alone results in a drop of only ∼30 mg of sodium consumed per day, which is too small for a clinical benefit to BP.^(31)^ Moreover, voluntary reformulation of packaged foods alone, even if achieved in other major contributors to dietary sodium in the NZ population (processed meats, sauces, condiments, cheese and soups and stocks),^(15)^ is insufficient to meet the WHO’s 2,000 mg/day sodium intake target.^(3)^ Thus, a national sodium reduction strategy incorporating all five activities of the WHO’s SHAKE package and clinical interventions, is strongly indicated for NZ; such a strategy should incorporate the recently released comprehensive 2024 WHO benchmarks^(32)^ and be designed to promote health equity. Concerning bread, stronger interventions from the WHO SHAKE package to Harness Industry and Adopt Standards for Labelling and Marketing are most relevant; these include making the HSR mandatory and the adoption of the WHO benchmarks by the HF.

### Conclusion

We found a modest decline in the sodium content of NZ packaged breads from 2013 to 2023, with more products meeting the HF (2024) and WHO (2021) sodium benchmarks over time. Findings imply that the two national initiatives, the HSR and HF reformulation programme, are gradually reducing sodium in NZ bread. However, further reductions across the packaged food supply, alongside a comprehensive national sodium reduction strategy, are required to meet the 2,000 mg WHO sodium reduction target.

## Supporting information

Tell et al. supplementary material 1 Tell et al. supplementary material

Tell et al. supplementary material 2 Tell et al. supplementary material

Tell et al. supplementary material 3 Tell et al. supplementary material

## Supplementary material

The supplementary material for this article can be found at https://doi.org/10.1017/jns.2025.10020
