# Supplementary material for: Changes in the sodium content of New Zealand packaged breads: 2013 to 2023
Source: J Nutr Sci. 2025 Jul 23;14:e52. doi: 10.1017/jns.2025.10020 (PMC12305275; doi:10.1017/jns.2025.10020)
Supplement: Tell et al. supplementary material 1 — Tell et al. supplementary material [file S2048679025100207sup001.docx]

¹ Data were removed due to issues such as duplicates, typing errors, missing ingredient lists, and the inclusion of products other than bread or bread products not classified under HF.

**Appendix 1.** Flow diagram for the process of identifying bread products for analysis through data cleaning.
