# Supplementary material for: Changes in the sodium content of New Zealand packaged breads: 2013 to 2023
Source: J Nutr Sci. 2025 Jul 23;14:e52. doi: 10.1017/jns.2025.10020 (PMC12305275; doi:10.1017/jns.2025.10020)
Supplement: Tell et al. supplementary material 2 — Tell et al. supplementary material [file S2048679025100207sup002.docx]

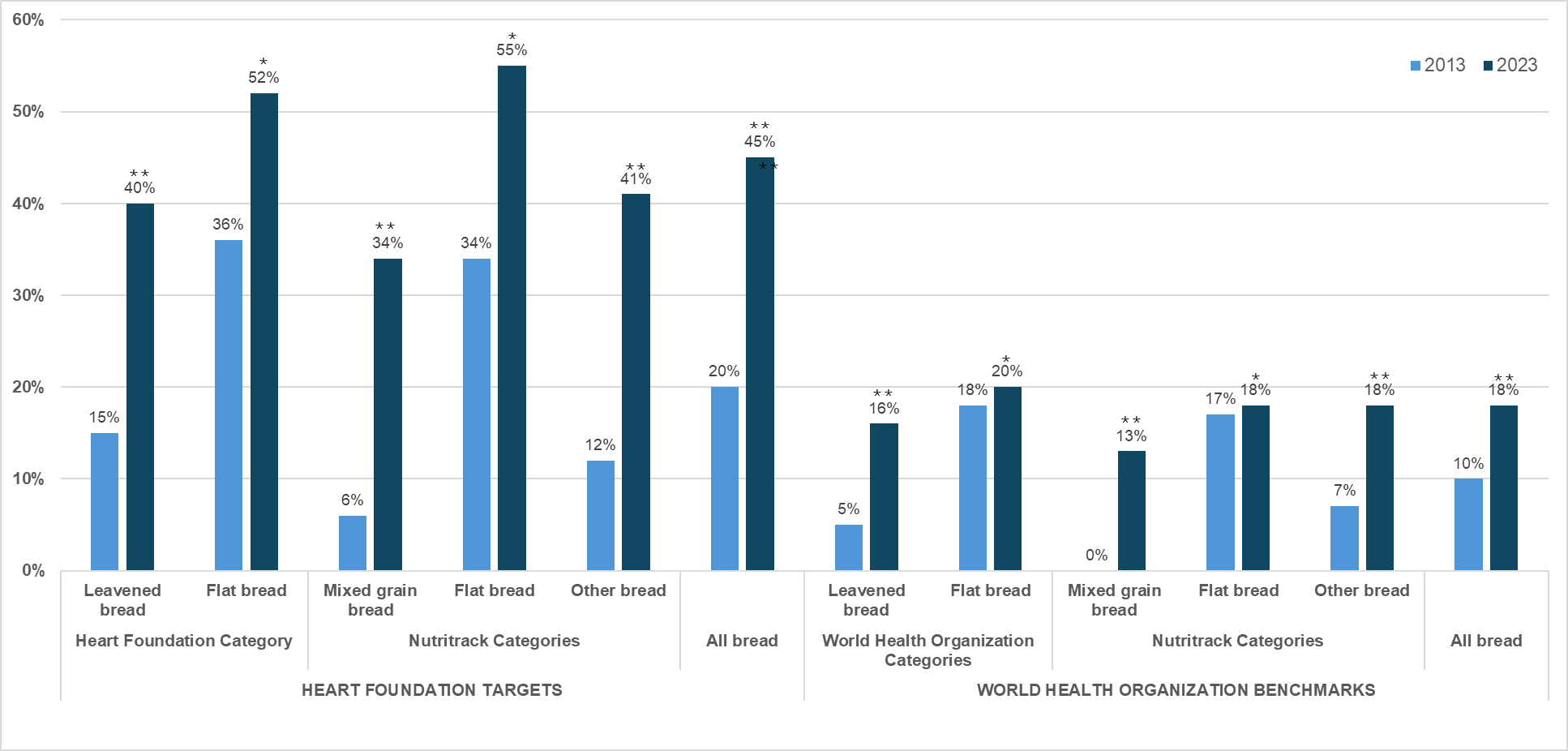


¹ The following bread categories are not presented as separate categories due to small product numbers (<30 for at least one year) making significance testing inappropriate: Nutritrack categories ‘Wholemeal bread’, ‘White bread’, ‘Fruit bread’, ‘Gluten-free flatbread’ and ‘Gluten-free Leavened bread’ and the WHO category ‘Sweet & raisin bread’. All categories are included in ‘All bread’. Statistically significant differences are indicated by a single asterisk (*) where *p* <0.05, and by two asterisks (**) where *p* <0.001.

**Appendix 2.** Percentage of breads¹ meeting the HF and WHO sodium benchmarks in 2013 and 2023.
