# Supplementary material for: Changes in the sodium content of New Zealand packaged breads: 2013 to 2023
Source: J Nutr Sci. 2025 Jul 23;14:e52. doi: 10.1017/jns.2025.10020 (PMC12305275; doi:10.1017/jns.2025.10020)
Supplement: Tell et al. supplementary material 3 — Tell et al. supplementary material [file S2048679025100207sup003.docx]

**Appendix 3.** Numbers and percentage of breads meeting sodium benchmarks in 2013 and 2023, overall and by category.

| **Bread category** | | **Year of data collection** | **Number of breads** | **HF Sodium Benchmark^1^** | **WHO Sodium Benchmark^2^** |
| --- | --- | --- | --- | --- | --- |
|  |  | **(y)** | **(n)** | **n (%)** | **n (%)** |
| **All bread** | | 2013 | 345 | 70 (20) | 33 (10) |
|  |  | 2023 | 309 | **138 (45) | **57 (18) |
| **HF bread category** | Leavened bread | 2013 | 261 | 40 (15) | - |
|  |  | 2023 | 196 | **79 (40) | - |
|  | Flat bread | 2013 | 84 | 30 (36) | - |
|  |  | 2023 | 113 | *59 (52) | - |
| **WHO bread category** | Leavened bread | 2013 | 245 | - | 12 (5) |
|  |  | 2023 | 186 | - | **29 (16) |
|  | Flat bread | 2013 | 84 | - | 15 (18) |
|  |  | 2023 | 112 | - | 22 (20) |
|  | Sweet & raisin bread | 2013 | 16 | - | 6 (38) |
|  |  | 2023 | 11 | - | 6 (55) |
| **Nutritrack bread category** | Wholemeal bread | 2013 | 16 | 3 (19) | 0 |
|  |  | 2023 | 11 | 4 (36) | 0 |
|  | Mixed grain bread | 2013 | 78 | 5 (6) | 0 |
|  |  | 2023 | 61 | **21 (34) | **8 (13) |
|  | White bread | 2013 | 35 | 0 | 0 |
|  |  | 2023 | 20 | 3 (15) | 1 (5) |
|  | Fruit bread | 2013 | 13 | 12 (92) | 6 (46) |
|  |  | 2023 | 10 | 10 (100) | 5 (50) |
|  | Flat bread | 2013 | 70 | 24 (34) | 12 (17) |
|  |  | 2023 | 83 | *46 (55) | 15 (18) |
|  | Gluten-free Flat bread | 2013 | 1 | 1 (100) | 0 |
|  |  | 2023 | 9 | 4 (44) | 4 (44) |
|  | Gluten-free leavened bread | 2013 | 32 | 13 (41) | 8 (25) |
|  |  | 2023 | 20 | 11 (55) | 7 (35) |
|  | Other bread | 2013 | 100 | 12 (12) | 7 (7) |
|  |  | 2023 | 95 | **39 (41) | *17 (18) |

¹ **HF Sodium Target:** Leavened bread (≤ 370 mg/100 g) and Flat bread (≤ 400 mg/100 g)

² **WHO Sodium Target:** Leavened bread (≤ 330 mg/100 g), Flat bread (≤ 320 mg/100 g) and Sweet and Raisin bread (≤ 310 mg/100 g)

Statistically significant differences are marked with one asterisk (*) for *p* <0.05 and with two asterisks (**) for *p* <0.001.
